# Indications, Complications, and Outcomes of Cardiac Surgery After Heart Transplantation: Results From the Cash Study

**DOI:** 10.3389/fcvm.2022.879612

**Published:** 2022-06-09

**Authors:** Johannes Gökler, Arezu Z. Aliabadi-Zuckermann, Alexandra Kaider, Amrut V. Ambardekar, Herwig Antretter, Panagiotis Artemiou, Alejandro M. Bertolotti, Udo Boeken, Vicens Brossa, Hannah Copeland, Maria Generosa Crespo-Leiro, Andrea Eixerés-Esteve, Eric Epailly, Mina Farag, Michal Hulman, Kiran K. Khush, Marco Masetti, Jignesh Patel, Heather J. Ross, Igor Rudež, Scott Silvestry, Sofia Martin Suarez, Amanda Vest, Andreas O. Zuckermann

**Affiliations:** ^1^Department of Cardiac Surgery, Medical University of Vienna, Vienna, Austria; ^2^Center for Medical Statistics, Informatics, and Intelligent Systems (CeMSIIS), Medical University of Vienna, Vienna, Austria; ^3^Division of Cardiology, University of Colorado School of Medicine, Aurora, CO, United States; ^4^Department of Cardiac Surgery, Medical University Innsbruck, Innsbruck, Austria; ^5^National Institute of Cardiovascular Diseases, Medical Faculty of the Comenius University, Bratislava, Slovakia; ^6^Heart and Lung Transplant Service, Favaloro Foundation University Hospital, Buenos Aires, Argentina; ^7^Department of Cardiac Surgery, Medical Faculty, Heinrich Heine University Hospital, Düsseldorf, Germany; ^8^Heart Transplant Division, Hospital Santa Creu i Sant Pau, Universitat Autònoma, Barcelona, Spain; ^9^Division Cardiac Surgery, Lutheran Hospital, Indiana University School of Medicine, Indiana, IA, United States; ^10^Complejo Hospitalario Universitario a Coruña (CHUAC), Centro de Investigación Biomédica en Red Enfermedades Cardiovasculares (CIBERCV), La Coruña, Spain; ^11^Department of Cardiac Surgery, University Hospital 12 de Octubre, Madrid, Spain; ^12^Heart and Heart-Lung Transplant Unit Medical, Department of Cardiovascular Surgery, Les Hôpitaux Universitaires NHC, Strasbourg, France; ^13^Department of Cardiac Surgery, Heidelberg University Hospital, Heidelberg, Germany; ^14^Division of Cardiovascular Medicine, Stanford University School of Medicine, Stanford, CA, United States; ^15^Heart Failure and Heart Transplant Unit, Istituto di Ricovero e Cura a Carattere Scientifico (IRCCS) Policlinico S. Orsola, Bologna, Italy; ^16^Heart Transplant Program, Cedars-Sinai Medical Center, Los Angeles, CA, United States; ^17^Cardiac Transplant Program, Peter Munk Cardiac Centre, Toronto, ON, Canada; ^18^Department of Cardiac Surgery, University Hospital Dubrava, Zagreb, Croatia; ^19^Thoracic Transplant Program, AdventHealth Transplant Institute, Florida, FL, United States; ^20^Cardiac Surgery Unit, Istituto di Ricovero e Cura a Carattere Scientifico (IRCCS) Policlinico S. Orsola, Bologna, Italy; ^21^Cardiac Transplantation Program, Tufts Medical Center, Boston, MA, United States

**Keywords:** cardiac transplantation, heart transplantation, cardiac surgery, heart failure, cardiac retransplantation

## Abstract

**Background:**

Allograft pathologies, such as valvular, coronary artery, or aortic disease, may occur early and late after cardiac transplantation. Cardiac surgery after heart transplantation (CASH) may be an option to improve quality of life and allograft function and prolong survival. Experience with CASH, however, has been limited to single-center reports.

**Methods:**

We performed a retrospective, multicenter study of heart transplant recipients with CASH between January 1984 and December 2020. In this study, 60 high-volume cardiac transplant centers were invited to participate.

**Results:**

Data were available from 19 centers in North America (*n* = 7), South America (*n* = 1), and Europe (*n* = 11), with a total of 110 patients. A median of 3 (IQR 2–8.5) operations was reported by each center; five centers included ≥ 10 patients. Indications for CASH were valvular disease (*n* = 62), coronary artery disease (CAD) (*n* = 16), constrictive pericarditis (*n* = 17), aortic pathology (*n* = 13), and myxoma (*n* = 2). The median age at CASH was 57.7 (47.8–63.1) years, with a median time from transplant to CASH of 4.4 (1–9.6) years. Reoperation within the first year after transplantation was performed in 24.5%. In-hospital mortality was 9.1% (*n* = 10). 1-year survival was 86.2% and median follow-up was 8.2 (3.8–14.6) years. The most frequent perioperative complications were acute kidney injury and bleeding revision in 18 and 9.1%, respectively.

**Conclusion:**

Cardiac surgery after heart transplantation has low in-hospital mortality and postoperative complications in carefully selected patients. The incidence and type of CASH vary between international centers. Risk factors for the worse outcome are higher European System for Cardiac Operative Risk Evaluation (EuroSCORE II) and postoperative renal failure.

## Introduction

Heart transplantation (HTX) confers excellent long-term survival in select patients with symptomatic end-stage heart failure. Cardiac surgery after heart transplantation (CASH) is a rarely used approach to improve allograft function and quality of life, and prolong survival (1), but has been described only in case reports and single-center experiences (2, 3). Although symptomatic tricuspid regurgitation is known as the most common cause for CASH (2), other valvular diseases, aortic pathology, and cardiac allograft vasculopathy (CAV) can occur in the HTX population (1–3). Coronary artery bypass grafting (CABG) is a safe surgical therapy for CAV, in selected cases, with acceptable long-term outcomes (1, 2). Furthermore, transcatheter and minimally invasive strategies to treat allograft pathologies have been reported with excellent short-term outcomes (4–9).

In this retrospective, multicenter, cohort study, we evaluated the safety of CASH and risk factors for subsequent morbidity and mortality.

## Materials and Methods

The study cohort was comprised of heart transplant recipients who underwent CASH between January 1984 and December 2020. CASH includes cardiac and aortic transcatheter interventions but does not include retransplantation, pacemaker/defibrillator placement, or reoperation due to bleeding complications. In total, 60 high-volume cardiac transplant centers performing more than 15 HTXs per year were invited to participate in the study. Each participating center obtained ethics approval from its institutional review board (Vienna ethics committee reference number: 1894/2017) and a data use agreement was executed with each center.

Relevant data on demographics, medical history, medications, surgeries, cardiac testing, outcomes, and complications were collected from the patients’ medical records and were de-identified by each center’s study coordinator. A password-protected, validated Excel file was sent to the primary investigator for statistical analysis. The European System for Cardiac Operative Risk Evaluation (EuroSCORE II) (10, 11), a validated scoring system that predicts the risk of in-hospital mortality after major cardiac surgery, was calculated.

The inverse Kaplan–Meier method was used to quantify median follow-up (12). To evaluate the effect of selected clinical factors on in-hospital mortality, we used univariate logistic regression models accounting for the center as a random effect. Survival after reoperation was described using the Kaplan–Meier method. Two-sided *p* < 0.05 were considered statistically significant. Statistical analyses were performed using SAS 9.4 (SAS Institute Inc., Cary, NC, United States).

## Results

### Study Population

Data from 110 patients were submitted by 19 centers (the United States-6; Spain-3; Austria-2; Germany-2; Argentina-1; Canada-1; Croatia-; France-1; Italy-1; and Slovakia-1) with a median of 3 (interquartile range [IQR] 2–8.5) patients per center; five centers reported ≥ 10 patients. The incidence of CASH was 0.86% (lowest 0.17%, highest 2.46%; and total number of HTX: 14,185). The median age at CASH was 57.7 (IQR 47.8–63.1) years with a median interval between HTX and CASH of 4.4 (IQR 1–9.6) years. Indications for surgery was valvular disease (*n* = 62), coronary artery disease (CAD) (*n* = 16), constrictive pericarditis (*n* = 17), aortic disease (*n* = 13), and myxoma (*n* = 2). Five patients (4.5%) had infectious etiology, including two fungal constrictive pericarditis, one aortic and one tricuspid valve endocarditis, and one infectious aortic pseudoaneurysm at the suture line. In 27 (24.5%) patients, CASH was performed in the first year, including 10 in the first 30 days. Thirty-six patients (32.7%) had an urgent indication for surgery. The surgical approaches were redo-sternotomy (*n* = 104), thoracotomy (*n* = 4), and transcatheter (TAVR; *n* = 2). Patient characteristics are provided in Table 1.

**TABLE 1:** Patient Characteristics, including postoperative details.

N	110
Sex (male), *n*	90 (82%)
Age at HTX, IQR, y	51.8 (40.7–57.5)
Donor age, IQR, y	42 (28–49)
EuroSCORE II, IQR	4.7 (2.9–8.2)
LVEF < 50%, *n*	13 (11.8%)
Diabetes, n	27 (24.5%), 14 NIDDM/13 IDDM
Creatinine pre-op (mg/dL), IQR	1.6 (1.3–2.2)
CKD (GFR < 60 mL/min), n	59 (53.6%), 5 dialysis dependent
CPB-time, IQR, min	109 (91.5–165)
Cross-clamp time, IQR, min	65 (46–94.5)
ICU stay, IQR, d	3.5 (1–6.3)
In-hospital stay, IQR, d	15 (10–24.5)
Perioperative complication, *n*	
Acute kidney injury	20 (18.2%)
Bleeding revision	10 (9.1%)
Pneumonia	8 (7.3%)
Wound infection	5 (4.5%)
Need for pacemaker	5 (4.5%)
Allograft failure	4 (3.6%)
Stroke	2 (1.8%)
In-hospital mortality, *n*	10 (9.1%)
1-year survival (SE)	86.2% (3.3)
Follow-up, IQR, y	8.2 (3.8–14.6)

*CKD, chronic kidney disease; CPB-time, cardiopulmonary bypass time; LVEF, left ventricular ejection fraction; GFR, glomerular filtration rate; HTX, heart transplantation; ICU, intensive care unit; IDDM, insulin-dependent diabetes mellitus; NIDDM, non-insulin-dependent diabetes mellitus; SE, standard error.*

### Outcome

The most common postoperative complication was acute kidney injury (Table 1). A permanent pacemaker was needed due to atrioventricular block in five patients, all after tricuspid valve surgery. Postoperative graft failure resulted in early death after tricuspid valve surgery in two patients and after pericardiectomy in one patient. In another patient, graft function recovered with postoperative extracorporeal membrane oxygenation (ECMO) support after total aortic arch replacement, aortic valve replacement, and CABG. Causes of death after discharge were cardiac (*n* = 14), infectious (*n* = 10), malignancy (*n* = 9), neurological complications (*n* = 3), and other (*n* = 8).

**FIGURE 1:**
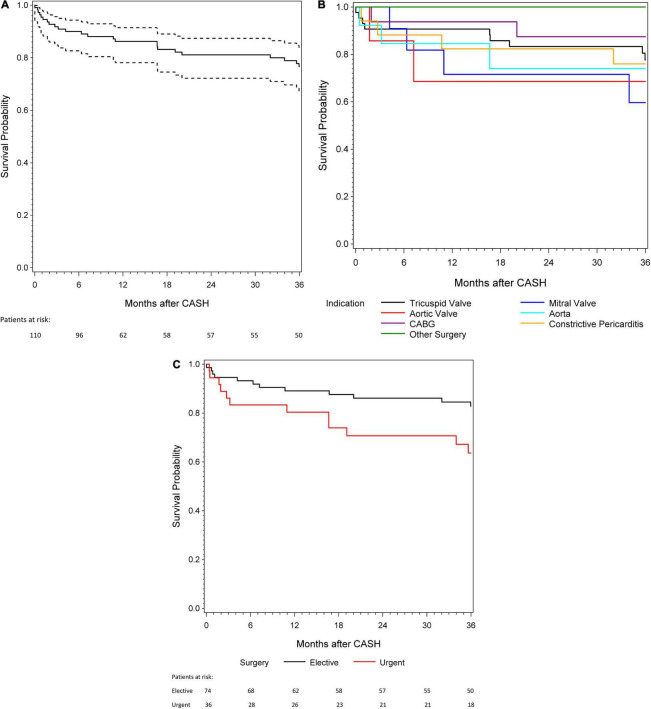
**(A)** A 3-year survival for patients with cardiac surgery after heart transplantation (CASH). **(B)** A 3-year survival according to the different indications for CASH. **(C)** A 3-year survival in patients with urgent and elective indication for CASH.

Overall survival was 86.2 ± 3.3 and 76.7 ± 4.2% after 1 and 3 years, respectively (Figure 1A). The 3-year survival stratified by indication for CASH is shown in Figure 1B. In-hospital mortality was 9.1% (*n* = 10), with systemic infection (*n* = 6), graft failure (*n* = 3), and bleeding (*n* = 1) as causes of death. Patients with urgent (compared with elective) CASH had worse in-hospital, 1-, and 3-year survival (Kaplan–Meier estimate: 83.3, 80.4, and 63.6% vs. 94.6, 89.1, and 82.8%, respectively; Figure 1C). In-hospital mortality was higher in patients with postoperative acute kidney injury (*n* = 20, 19.1%; 35.0 vs. 3.5% for urgent vs. elective, respectively). In univariate logistic regression analysis, postoperative acute kidney injury (odds ratio [*OR*] 14.7 [95% confidence interval [*CI*] 3.3–65.6], *p* = 0.0006) and higher EuroSCORE II (*OR* for 2-fold increase 2.0 [1.0–3.7], *p* = 0.04) were statistically significantly associated with in-hospital mortality. Higher in-hospital mortality was associated with urgent indication for surgery (*OR* 3.5 [0.9–13.6], *p* = 0.07), as well as older age at the time of reoperation (*OR* for 10-year increase 1.8 [0.9–3.6], *p* = 0.08). However, these effects were not statistically significant. Heart transplant recipient sex (*p* = 0.32), time since HTX (*p* = 0.89), and baseline serum creatinine (*p* = 0.55) were not statistically significantly associated with in-hospital mortality in univariate logistic regression models (Supplementary Table 1).

### Valvular Disease

#### Tricuspid Valve Surgery

Tricuspid valve surgery was the most common CASH (*n* = 48) at 14 centers (Table 2): 7 centers with one patient, 4 centers with two, and 4 centers with more than two (*n* = 3, 8, 10, and 12). Of these 48 patients, 5 had tricuspid valve surgery in combination with surgical procedures involving the mitral valve (*n* = 3), aortic valve (*n* = 1), and aorta (*n* = 1).

**TABLE 2:** Tricuspid valve surgery.

N	43
Sex (male), *n*	34 (79.1%)
Age at CASH, y	52.0 (35.4–63.4)
EuroSCORE II	4.4 (2.9–6.3)
LVEF < 50%, *n*	7 (16.3%)
Creatinine pre-op (mg/dL)	1.7 (1.5–2.3)
CKD (GFR < 60 mL/min), *n*	21 (48.8%), 2 dialysis dependent
Time to CASH, y	3.8 (0.9–9.8)
Urgent operation, *n*	14 (32.6%)
Indication, *n*	
Biopsy induced	22
Annular dilation	8
Degenerative	4
Pacemaker lead	1
Endocarditis	1
Unknown cause	7
Operation, *n*	
Repair	22 (51.2%)
biological valve/mechanical valve	19 (44.2%)/2 (4.7%)
Access (sternotomy), *n*	42; thoracotomy *n* = 1
ICU stay, d	3.5 (2–8)
Complications, *n*	
Renal replacement therapy	11 (26.2%)
AVB, PM-implant	5 (11.9%)
Bleeding revision	5 (11.9%)
Right heart failure	2 (4.7%)
Pneumonia	4 (9.3%)
Wound infection	1
Dissection at cannulation site	1
Recurrent severe regurgitation	1 (biological valve replacement)
In-hospital mortality, *n*	4 (9.3%)
1-year survival (SE)	90.7% (9.3)

*AVB, atrioventricular block; CASH, cardiac surgery after heart transplantation; CKD, chronic kidney disease; LVEF, left ventricular ejection fraction; GFR, glomerular filtration rate; ICU, intensive care unit; PM-implant, pacemaker implantation; SE, standard error.*

For isolated tricuspid valve surgeries, the indication was severe, symptomatic tricuspid regurgitation. Biopsy-induced tricuspid regurgitation was the most common indication for surgery. Twelve patients (27.9%) underwent CASH in the first year post-transplant, most within the first 90 days (*n* = 9).

Major complications were comprised of intraoperative aortic dissection at the cannulation site, which required hemiarch replacement in one patient. Two patients developed intraoperative right heart failure, and both died perioperatively. One patient underwent biological tricuspid valve replacement 4 months after repair due to severe recurrent regurgitation. Four early deaths occurred on postoperative days (PODs) 0, 12, 26, and 34.

#### Mitral Valve Surgery

Five centers reported 12 patients who had mitral valve surgery (Table 3). One patient with concomitant mitral and aortic valve replacement and tricuspid valve reconstruction is described in the aortic valve surgery section. Three patients had tricuspid valve surgery (2 repairs; 1 replacement) as a concomitant procedure. The median age at CASH was 61.5 (IQR 51.6–62.6) years, and the time to CASH was 7.2 (IQR 3.1–10.1) years. One procedure was performed on postoperative day (POD) 2 due to severe mitral regurgitation with a ruptured cord in the anterior leaflet. Surgical access was usually sternotomy, except for one patient who had a thoracotomy. The median postoperative intensive care unit (ICU) stay was 5 (IQR 1–8) days and the in-hospital stay was 15 (IQR 10–21.8) days. No early perioperative (in-hospital) or surgery-related deaths occurred. Postoperative complications were acute kidney injury and reintubation in one patient. Furthermore, 1-year survival was 71.6%, and death was not related to the mitral valve surgery.

**TABLE 3:** Mitral valve surgery.

Pt	Sex	Age	Euro SCORE II	LVEF < 50%	Creatinine	GFR < 60 ml/min	Time to CASH, y	Urgency	Indication	Pathology	Operation	Mortality	Follow-up, y
1	m	47.8	10.27	−	1.3	−	2 days	Urgent	Ruptured chord	Regurgitation	Biological valve	−	5.7
2	f	66.6	6.93	n.a.	2.7	yes	0.6	Elective	Annular dilation	Regurgitation	Repair	−	1.2
3	m	65.1	9.53	yes	1.6	yes	3	Urgent	Degenerative	Regurgitation	Mechanical valve	Cardiac	2.8
4	f	53.1	10.43	−	4	dialysis	3.2	Urgent	Degenerative	Regurgitation	Mechanical valve	Other	0.9
5	m	61.6	2.89	−	1.1	−	5.1	Elective	Degenerative	Regurgitation	Mechanical valve	Other	14.4
6	m	63.7	3.05	−	1.3	−	7.2	Elective	Degenerative	Regurgitation	Mechanical valve	Other	12.3
7	m	61.5	4.8	−	2.5	yes	7.8	Elective	Degenerative	Regurgitation	Repair	Infection	0.4
8	f	50.2	3.42	yes	n.a.	−	9.6	Elective	Degenerative	Both	Mechanical valve	−	0.7
9	m	61.5	8.22	n.a.	3.4	yes	10.6	Elective	Degenerative	Stenosis	Mechanical valve	MOF	5.1
10	m	61.5	5.67	−	1.2	−	10.8	Urgent	Degenerative	Regurgitation	Mechanical valve	Other	9.3
11	m	26.3	3.46	−	0.7	−	13.4	Elective	Degenerative	Regurgitation	Repair	n.a.	0.5
med		61.5 (51.6–62.6)	5.7 (3.4–8.9)		1.5 (1.2–2.7)		7.2 (3.1–10.1)						

*CASH, cardiac surgery after heart transplantation; Creatinine, Creatinine pre-op (mg/dL); LVEF, left ventricular ejection fraction; GFR, glomerular filtration rate; ICU, intensive care unit; n.a., not available; MOF, multi-organ failure; pt, patient.*

#### Aortic Valve Surgery

Cardiac surgery after heart transplantation due to aortic valve disease was performed in 7 patients at six centers (Table 4). The median age and allograft age at CASH were 61.3 (IQR 56.9–68.7) and 59.2 (IQR 45.8–70.6) years, respectively. One patient underwent CASH in the first year (POD 20, unknown cause of aortic regurgitation), and the median time to CASH was 7.1 (IQR 3.7–10.6) years. Surgical access was sternotomy in all but two patients who had transcatheter aortic valve replacement (TAVR). The postoperative ICU stay was 4 days (IQR 1.8–6.3), and the in-hospital stay was 18 days (11–34). Two patients had a complicated postoperative course requiring surgical revision (bleeding) and died 52 and 219 days after CASH, respectively; all the others are still alive. Four patients underwent aortic valve surgery due to aortic disease (aneurysm/dissection) and are described in the aortic surgery section.

**TABLE 4:** Aortic valve surgery.

Pt	Sex	Age	Euro SCORE II	Creatinine	Time to CASH, y	Urgency	Pathology	Operation	Concomitant procedure	Complications	Mortality	Follow-up, y
1	m	54.4	16.5	1.4	0.1	Urgent	Regurgitation	Mechanical valve	CABG	Bleeding, RRT, stroke, pneumonia	MOF	0.1
2	m	52.9	6.7	1.2	2.0	Urgent	Endocarditis	Biological valve			−	7.7
3	m	59.3	2.7	n.a.	5.3	Elective	Regurgitation	Mechanical valve			−	0.5
4	m	62.3	15.8	4.4	7.1	Urgent	Stenosis	Mechanical valve	Mitral + tricuspid	RRT	−	0.9
5	m	61.3	2.8	1.7	9.7	Elective	Stenosis	TAVR, femoral			−	3.8
6	m	75.1	7.0	2.1	11.4	Elective	Stenosis	Biological valve		Bleeding	Bleeding	0.6
7	m	83.2	5.1	1.0	22.9	Elective	Stenosis	TAVR, apical			−	2.5
med		61.3 (56.9–68.7)	6.7 (4.0–11.4)	1.5 (1.3–2.0)	7.1 (3.7–10.6)							

*CABG, coronary artery bypass graft; CASH, cardiac surgery after heart transplantation; ICU, intensive care unit; n.a not available; MOF, multi-organ failure; MV, mitral valve; pt, patient; RRT, renal replacement therapy.*

### Aortic Surgery

Aortic surgery due to ascending aortic pathologies (dissection, aneurysm, or pseudoaneurysm) was performed in 13 patients at 11 centers (Table 5). The median age at CASH was 57.5 (IQR 50.3–61.1) years and the time to CASH was 3.9 (IQR 0.8–7.6) years. Four patients had surgery within the first year (PODs 20, 50, 110, and 235). Allograft function was preserved in all patients. The median preoperative serum creatinine was 1.1 (1.0–1.7) mg/dl. Two patients had reduced kidney function preoperatively, including one patient who was dialysis-dependent.

**TABLE 5:** Aortic surgery.

Pt	Sex	Age	Euro SCORE II	Time to CASH, y	Urgency	Indication	Operation	Concomitant Procedure	Complications	Mortality	Follow-up, y
1	m	58.6	9.4	20 days	Urgent	Dissection	Bentall, mechanical			−	8.5
2	m	57.1	21.2	0.1	Urgent	Pseudoaneurysm	Supracommissural replacement	TV repair	infection	MOF	0.3
3	m	61.5	10.4	0.3	Urgent	Pseudoaneurysm	Supracommissural replacement			−	2.6
4	m	39.8	7.1	0.8	Elective	Pseudoaneurysm	Supracommissural replacement			−	5.4
5	m	58.0	13.1	1.3	Elective	Aneurysm	Total arch replacement	CABG, aortic valve, stentgraft	ECMO	−	0.9
6	m	61.1	28.3	2.4	Urgent	Pseudoaneurysm	Supracommissural replacement		Bleeding	Bleeding	21 days
7	f	53.8	9.1	3.9	Urgent	Dissection	Supracommissural replacement			Cardiac	5.7
8	m	50.3	23.3	5.1	Urgent	Dissection	Supracommissural replacement	CABG, aortic valve		−	4.0
9	m	57.5	8.7	7.1	Urgent	Aneurysm	Hemiarch replacement	CABG		−	0.3
10	m	48.7	3.8	7.6	Elective	Pseudoaneurysm	Supracommissural replacement			Stroke	3.9
11	f	61.6	6.7	7.7	Elective	Aneurysm	Supracommissural replacement			Cardiac	4.2
12	f	32.7	13.8	10.0	Urgent	Aneurysm	Bentall, biological	CABG		−	1.1
13	m	72.3	31.3	18.1	Urgent	Dissection	Supracommissural replacement			Malignancy	1.4
med		57.5 (50.3–61.1)	10.4 (8.7–21.2)	3.9 (0.8–7.6)							

*CABG, coronary artery bypass graft; CASH, cardiac surgery after heart transplantation; ICU, intensive care unit; MOF, multi-organ failure; pt, patient; TV, tricuspid valve.*

The median postoperative ICU stay was 5 (IQR 4–6) days, and the in-hospital stay was 18 (IQR 14–24) days. One patient required temporary ECMO due to postoperative stunning and allograft function subsequently recovered. Early postoperative death (PODs 14 and 97) occurred in two patients after a complicated clinical course. The 1-year survival was 84.6%.

### Coronary Artery Bypass Grafting Surgery

Sixteen patients (87.5% male) from eight centers who had CAD as the indication for CABG surgery are described in Table 6. The median age and allograft age at CASH were 60.3 (IQR 54.6–63.3) and 45.8 (IQR 36.9–54.3) years, respectively. The time to CASH was 8.8 (IQR 5.9–9.8) years. Allograft function, as measured by the left ventricular ejection fraction (LVEF), was severely impaired (< 30%) in two patients, preserved (≥ 50%) in seven patients, and data were not available in seven patients. Eleven patients had reduced kidney function preoperatively, including a patient who was dialysis-dependent.

**TABLE 6:** Coronary artery bypass graft surgery.

Pt	Sex	Age	Euro SCORE II	Time to CASH, y	Urgency	Indication	Operation	OP-details	Complications	Mortality	Follow-up, y	PCI/HTX after CASH
1	m	63.0	6.8	3 days	Urgent	Donor stenosis	Vein RCA	RVAD 13d		−	12.3	−
2	m	51.8	2.0	3.6	Elective	CAV	LIMA LAD			Cardiac	5.9	PCI
3	f	62.4	11.5	4.0	Urgent	Iatrogenic dissection	Vein LAD + CX		Sepsis	MOF	0.2	PCI
4	m	59.5	3.7	5.4	Urgent	CAV	LIMA LAD, Vein CX			−	3.8	−
5	m	52.9	2.7	6.1	Elective	CAV	LIMA CX, RIMA DG			Cardiac	9.1	−
6	m	57.4	4.6	7.8	Elective	CAV	Vein LAD + CX			Tumor	7.1	PCI
7	m	65.6	5.5	7.8	Elective	CAV	LIMA LAD, RIMA RCA, Vein CX			−	16.6	PCI
8	m	62.4	4.0	8.5	Elective	CAV	LIMA LAD, RIMA CX			Tumor	7.1	−
9	m	71.4	3.7	9.0	Elective	CAV	LIMA LAD, RIMA RCA (T-graft)			Tumor	8.5	−
10	m	64.3	8.7	9.3	Elective	CAV	LIMA LAD, RIMA DG, Radial CX		Sternal infection	Cardiac	7.6	−
11	m	55.2	2.7	9.3	Elective	CAV	LIMA LAD	off-pump		−	12.3	PCI
12	f	59.6	2.5	9.6	Elective	CAV	LIMA LAD, Vein CX			−	11.8	PCI, Re-HTX
13	m	60.9	2.8	10.4	Elective	CAV	LIMA LAD	MID-CAB		Cardiac	13.3	PCI
14	m	38.4	8.1	10.6	Elective	CAV	LIMA LAD	MID-CAB	RRT	Infection	1.7	−
15	m	48.3	2.7	12.0	Elective	CAV	LIMA LAD + DG, RIMA CX, Vein RCA			−	19.2	PCI
16	m	69.8	29.5	16.2	Urgent	CAV	LIMA LAD, Vein CX + DG + RCA			−	3.9	−
med		60.3 (54.6–63.3)	3.9 (2.7–7.1)	8.8 (5.9–9.8)								

*CABG, coronary artery bypass graft; CAV, cardiac allograft vasculopathy; CASH, cardiac surgery after heart transplantation; CX, circumflex artery; DG, diagonal branch; ICU, intensive care unit; LIMA, left internal mammary artery; n.a., not available; MID-CAB, minimally invasive direct coronary artery bypass; MOF, multi-organ failure; PCI, percutaneous coronary intervention; pt, patient; re-HTX, cardiac retransplantation; RIMA, right internal mammary artery; RVAD.*

One patient had percutaneous coronary intervention (PCI) in the allograft prior to CABG surgery. Four patients had diabetes mellitus, including two patients who were insulin-dependent. Coronary angiography was performed in all of the planned CABG surgeries, and computed tomography (CT) was available in only half the patients.

The indications for CASH were CAV (*n* = 14), iatrogenic left main stem dissection following routine coronary angiography (*n* = 1), and right coronary artery stenosis unknown at the time of HTX (CABG performed on POD 3). Surgical access was median re-sternotomy in all but two patients who underwent minimally invasive direct coronary artery bypass (MIDCAB). The left internal mammary artery was used as the bypass graft in 81.3% of procedures, and the right internal mammary artery in 37.5%. Saphenous vein grafts were used in half of the patients, radial artery only in one patient.

The median postoperative ICU stay was 2.5 (IQR 1–3.8) days and the in-hospital stay 12.5 (IQR 11–20.8) days. The 1-year survival was 93.8% with only one CASH-related death (POD 59, after iatrogenic left main stem dissection). Due to progression of CAV, half of the patients subsequently had PCI with drug-eluting stents and one patient underwent cardiac retransplantation.

In six additional patients, CABG surgery was a concomitant procedure, and the patients are described in the aortic valve surgery (*n* = 2), aortic surgery (*n* = 3), and constrictive pericarditis (*n* = 1) sections.

### Pericardiectomy

Five centers reported 17 patients who had pericardiectomy due to constrictive pericarditis (Table 7). Three centers reported more than one case each (*n* = 4, 5, and 6). The median age at CASH was 56.3 (IQR 54.7–63.5) years and the time to CASH was 1.7 (0.7–3.1) years. Five patients underwent pericardiectomy in the first year. One patient was on vasopressor support with an urgent indication for surgery. Median preoperative serum creatinine was 1.6 (1.5–2.2) mg/dl. Kidney function was reduced preoperatively [glomerular filtration rate (GFR) < 60 ml/min] in 58.8% of patients, and none were dialysis-dependent. Access was *via* a median re-sternotomy for extensive pericardiectomy. The median ICU stay was 1.5 (IQR 1–2.5) days and in-hospital stay was 15 (IQR 8–19) days. In one patient, intraoperative injury of the left anterior descending artery resulted in anastomosis of a vein bypass graft. Reintubation was required for pneumonia in one patient, and another patient had a deep sternal infection. One patient had fatal postoperative right heart failure. Overall 1-year survival was 82.4%, and two early deaths were reported (PODs 21 and 83).

**TABLE 7:** Pericardiectomy.

Pt	Sex	Age	Euro SCORE II	Time to CASH, y	Urgency	Complications	Mortality	Follow-up, y
1	m	61.5	2.1	0.2	Urgent		n.a.	2.7
2	m	63.5	4.3	0.2	Elective		Cardiac	0.9
3	m	55.1	4.7	0.3	Elective		−	9.1
4	m	57.4	4.7	0.5	Elective		−	1.5
5	m	65.7	7.7	0.7	Elective	RRT	−	6.9
6	f	47.5	3.4	1.0	Elective	Infection	−	8.2
7	m	63.6	5.2	1.5	Elective		Malignancy	3.5
8	m	45.7	2.7	1.6	Elective		n.a.	4.4
9	m	64.8	3.1	1.7	Elective		Infection	3.5
10	m	56.3	4.7	2.6	Elective		−	0.2
11	m	31.8	2.7	3.0	Elective		Infection	4.2
12	m	26.3	4.7	3.1	Elective		−	9.1
13	m	61.6	2.9	3.1	Elective		n.a.	3.5
14	m	55.9	2.7	3.5	Elective	Right heart failure	Cardiac	0.1
15	f	54.7	4.6	4.3	Elective	Infection, RRT	MOF	0.2
16	m	69.4	4.7	8.3	Urgent		Malignancy	14.1
17	m	55.5	2.7	17.3	Elective		−	3.7
med		56.3 (54.7–63.5)	4.3 (2.7–4.7)	1.7 (0.7–3.1)				

*CASH, cardiac surgery after heart transplantation; FU, follow-up; ICU, intensive care unit; n.a., not available; MOF, multi-organ failure; pat, patient; RRT, renal replacement therapy.*

### Other Operations

Left atrial myxoma resection was performed 1.7 and 14.3 years after HTX (Table 8). Both had an uneventful postoperative course and no recurrent disease.

**TABLE 8:** Other operations.

	Patient 1	Patient 2	Patient 3
Indication	Pulmonary valve regurgitation	Myxoma	Myxoma
CASH	Biological valve	Resection	Resection
Sex	Male	Female	Male
Age	36.1	68	28.5
EuroSCORE II	7.8	9.9	2.7
Time to CASH, y	7 days	1.7	14.3
Urgency	Urgent	Elective	Elective
Complications	Bleeding, pneumonia		
Cause of death	−	−	−
Follow-up, y	3.8	0.7	5

*CASH, cardiac surgery after heart transplantation.*

One patient underwent biological pulmonary valve replacement due to regurgitation (valve injury at the time of procurement/implantation) on POD 7. After a complicated postoperative course with surgical revision due to bleeding and pneumonia, the patient is alive 3.8 years after CASH without prosthesis degeneration.

## Discussion

Our multicenter study describes the largest cohort of patients with CASH worldwide. We demonstrate that CASH is an acceptable therapy for different cardiovascular pathologies early and late after HTX. Overall in-hospital and 1-year mortality after CASH were acceptable (9.1 and 13.8%, respectively). Urgent indication for CASH, such as endocarditis, infected pseudoaneurysm, aortic dissection, and iatrogenic complications, was strikingly, but not statistically significantly, associated with higher in-hospital mortality. These indications are also associated with high mortality in the general heart surgery population, and in previous reports on CASH (2, 3). Postoperative acute kidney injury requiring renal replacement therapy was the most common complication after CASH and was associated with higher in-hospital mortality. Age at time of CASH and higher EuroSCORE II were also associated with higher in-hospital mortality. However, the effect of age was not statistically significant.

The incidence of postoperative systemic infection and deep sternal wound infection was low (13, 14). Reduction or discontinuation of mammalian target of rapamycin (mTOR) inhibitors several weeks to months prior to elective CASH may be considered due to reports of impaired wound healing (15).

Severe tricuspid regurgitation is the most common reason for CASH (16). HTX-specific tricuspid valve pathologies are biopsy-induced injury to the chordae (leaflets), ischemic injury to the papillary muscle as a consequence of CAV or rejection, and distortion of the valvular apparatus [biatrial implantation technique (2, 17–24). Endocarditis is rare, but is more common than in the general population due to increased risk arising from immunosuppression and frequent central venous access, such as endomyocardial biopsies (25).

Indications for surgery must be carefully considered, especially in patients with ventricular dysfunction and/or pulmonary hypertension because they are at risk of right ventricular failure after CASH. Potential underlying disease processes, such as CAV or acute rejection must be ruled out prior to CASH.

Tricuspid valve repair with annuloplasty should only be performed in HTX patients with annular dilation; valve replacement is recommended in complex valvular pathologies or residual regurgitation after repair (21). Besides the risks associated with life-long anticoagulation, mechanical valve replacement rules out the right-sided endomyocardial biopsy. Interventional edge-to-edge repair of the tricuspid valve using the MitraClip system has been reported with perioperative success (26), but long-term data are lacking.

Mitral valve surgery after HTX has been described rarely. Pathology can be related to annular dilation or degeneration of the leaflets or papillary muscles, typically as a consequence of CAV or acute rejection, often accompanied by ventricular dysfunction (27–29). Iatrogenic injury after left-sided endomyocardial biopsy may lead to acute mitral regurgitation. Mitral stenosis has been described in dialysis-dependent HTX patients in association with hyperparathyroidism (30). Mitral valve replacement may be preferred over repair due to complex valvular pathologies in patients with HTX, and to achieve shorter cardiopulmonary bypass times in patients with ventricular dysfunction. In three of our patients, concomitant tricuspid valve repair or replacement was performed without perioperative complications, but postprocedural death occurred 0.5, 0.9, and 2.8 years after CASH.

Minimally invasive CASH *via* thoracotomy confers the advantage of avoiding resternotomy. Transcatheter interventions may be reasonable for patients at high surgical risk with appropriate valvular pathology, but the atrial and atrio-ventricular anatomy can be challenging due to distortions after HTX (9).

Cardiac surgery after heart transplantation due to symptomatic degenerative aortic stenosis in the allograft may occur more often with the acceptance of marginal donor hearts. Single-center case reports have reported acceptable perioperative outcomes (16, 31–33), and case reports on TAVR in HTX patients with high surgical risk have presented data on favorable short-term outcomes (4–8); however, long-term data, as well as data are data on the durability of biological and mechanical valves, are lacking. Aortic valve endocarditis after HTX is extremely rare (34). Our cohort included a patient with biological valve replacement due to endocarditis and 7.7 years of follow-up without valvular degeneration.

Aortic surgery was the most heterogeneous group in our series. Case reports have described successful CASH for ascending aortic aneurysm, dissection, or pseudoaneurysm of the aortic anastomosis (1–3, 35). Aortic pathologies after HTX typically arise at the site of aortic anastomosis due to flow turbulence (donor/recipient aortic size mismatch), infection, or hypertension (36). Due to urgency, the precise preoperative planning of surgery is limited in acute type A dissection and infected pseudoaneurysm. Our data are in line with previously published data (1). Two patients with urgent surgery due to infected pseudoaneurysm had early surgery-related deaths, highlighting the high risk of mortality associated with this rare disease (36).

Surgical revascularization for CAV is safe, with acceptable long-term results, in HTX patients with acceptable coronary anatomy (type A lesion, Stanford Classification), and elective indication for surgery (1–3, 37). This approach is generally limited, however, by the diffuse nature of CAV (37, 38) and inexorable disease progression in most patients (3, 39), which may necessitate additional interventions (3, 40). The patency of arterial grafts is superior to vein grafts in CASH, with patency of the internal thoracic artery reported up to 20 years (3, 41). Though the left internal thoracic artery was used in most of our patients, the radial artery had good mid-term results in a published case series and may be an adjunct graft in patients with CABG prior to HTX (3).

Constrictive pericarditis after HTX is rare and is typically associated with recurrent pericardial effusions, allograft rejection, or biopsy-related complications (42–44). As most of the participating centers did not report any cases of constrictive pericarditis requiring pericardiectomy, we cannot draw conclusions as to whether patients were undiagnosed at some centers and whether surgical and/or treatment strategies differed at the three centers that reported cases of constrictive pericarditis.

This study is limited by its retrospective nature, the small numbers for each procedure type, and the fact that participation was by invitation only—not all eligible heart transplant centers reported data. It may be of interest, however, for multinational heart transplant registries to begin collecting data on CASH, given the growing population of heart transplant recipients worldwide with improved long-term survival and the increasing use of minimally invasive surgical and transcatheter approaches.

We conclude that CASH is generally safe, with low in-hospital mortality and postoperative complications in carefully selected patients. Nevertheless, it is rarely performed, with differences in practice between heart transplant centers worldwide. Higher EuroSCORE II and postoperative acute kidney injury are associated with higher in-hospital mortality.

## Data Availability Statement

The raw data supporting the conclusions of this article will be made available by the authors, without undue reservation.

## Author Contributions

JG and AA-Z designed the study and wrote the manuscript. JG organized the database. AK performed the statistical analysis. All authors contributed to manuscript revision and approved the submitted version.

## Conflict of Interest

The authors declare that the research was conducted in the absence of any commercial or financial relationships that could be construed as a potential conflict of interest.

## Publisher’s Note

All claims expressed in this article are solely those of the authors and do not necessarily represent those of their affiliated organizations, or those of the publisher, the editors and the reviewers. Any product that may be evaluated in this article, or claim that may be made by its manufacturer, is not guaranteed or endorsed by the publisher.

## Abbreviations

BiVAD: biventricular assist device
CABG: coronary artery bypass grafting
CAV: cardiac allograft vasculopathy
CASH: cardiac surgery after heart transplantation
CI: confidence interval
ECMO: extracorporeal membrane oxygenation
EuroSCORE II: European System for Cardiac Operative Risk Evaluation
GFR: glomerular filtration rate
HTX: heart transplantation
IQR: interquartile range
LVEF: left ventricular ejection fraction
OR: odds ratio
PCI: percutaneous coronary intervention
POD: postoperative day
TAVR: transcatheter aortic valve replacement.

## References

[1] Holmes TR Jansz PC Spratt P Macdonald PS Dhital K Hayward C Cardiac surgery is successful in heart transplant recipients. *Heart Lung Circ.* (2014) 23:703–10. 10.1016/j.hlc.2014.03.003 24709393

[2] Goerler H Simon A Warnecke G Meyer AL Kuehn C Haverich A Cardiac surgery late after heart transplantation: a safe and effective treatment option. *J Thorac Cardiovasc Surg.* (2010) 140:433–9. 10.1016/j.jtcvs.2010.02.033 20381816

[3] Goekler J Zuckermann A Osorio E Brkic FF Uyanik-Uenal K Laufer G Cardiac surgery after heart transplantation: elective operation or last exit strategy? *Transplant Direct.* (2017) 3:e209. 10.1097/TXD.0000000000000725 29138760 PMC5627740

[4] Zanuttini D Armellini I Bisceglia T Spedicato L Bernardi G Muzzi R Transcatheter aortic valve implantation for degenerative aortic valve regurgitation long after heart transplantation. *Ann Thorac Surg.* (2013) 96:1864–6. 10.1016/j.athoracsur.2013.03.040 24182478

[5] Bruschi G De Marco F Oreglia J Colombo P Moreo A De Chiara B Transcatheter aortic valve implantation after heart transplantation. *Ann Thorac Surg.* (2010) 90:e66–8. 10.1016/j.athoracsur.2010.08.021 20971222

[6] Chandola R Cusimano R Osten M Horlick E. Postcardiac transplant transcatheter core valve implantation for aortic insufficiency secondary to impella device placement. *Ann Thorac Surg.* (2012) 93:e155–7. 10.1016/j.athoracsur.2011.12.025 22632535

[7] De Praetere H Ciarka A Dubois C Herijgers P. Transapical transcatheter aortic valve implantation in a heart transplant recipient with severely depressed left ventricular function. *Interact Cardiovasc Thorac Surg.* (2013) 16:906–8. 10.1093/icvts/ivt048 23460597 PMC3653466

[8] Ahmad K Terkelsen CJ Terp KA Mathiassen ON Norgaard BL Andersen HR Transcatheter aortic valve implantation in a young heart transplant recipient crossing the traditional boundaries. *J Thorac Dis.* (2016) 8:E711–4. 10.21037/jtd.2016.07.61 27621906 PMC4999735

[9] Ferraro P Biondi-Zoccai G Giordano A. Transcatheter mitral valve repair with mitraclip for significant mitral regurgitation long after heart transplantion. *Catheter Cardiovasc Interv.* (2016) 88:144–9. 10.1002/ccd.26153 26333048

[10] Nashef SA Roques F Sharples LD Nilsson J Smith C Goldstone AR EuroSCORE II. *Eur J Cardiothorac Surg.* (2012) 41:734–44discussion744–5. 10.1093/ejcts/ezs043 22378855

[11] Garcia-Valentin A Mestres CA Bernabeu E Bahamonde JA Martin I Rueda C Validation and quality measurements for EuroSCORE and EuroSCORE II in the Spanish cardiac surgical population: a prospective, multicentre study. *Eur J Cardiothorac Surg.* (2016) 49:399–405. 10.1093/ejcts/ezv090 25762397

[12] Schemper M Smith TL. A note on quantifying follow-up in studies of failure time. *Control Clin Trials.* (1996) 17:343–6. 10.1016/0197-2456(96)00075-x 8889347

[13] Filsoufi F Rahmanian PB Castillo JG Pinney S Broumand SR Adams DH. Incidence, treatment strategies and outcome of deep sternal wound infection after orthotopic heart transplantation. *J Heart Lung Transplant.* (2007) 26:1084–90. 10.1016/j.healun.2007.07.036 18022072

[14] Zuckermann A Barten MJ. Surgical wound complications after heart transplantation. *Transpl Int.* (2011) 24:627–36. 10.1111/j.1432-2277.2011.01247.x 21418335

[15] Nashan B Citterio F. Wound healing complications and the use of mammalian target of rapamycin inhibitors in kidney transplantation: a critical review of the literature. *Transplantation.* (2012) 94:547–61. 10.1097/TP.0b013e3182551021 22941182

[16] Kpodonu J Massad MG Geha AS. Surgical considerations in the correction of valve dysfunction following heart transplantation. *Clin Transplant.* (2005) 19:694–7. 10.1111/j.1399-0012.2004.00316.x 16146564

[17] Locali RF Matsuoka PK Cherbo T Gabriel EA Buffolo E. [Should biatrial heart transplantation still be performed?: a meta-analysis]. *Arq Bras Cardiol.* (2010) 94:829–40. 10.1590/s0066-782x2010000600018 20625642

[18] Sun JP Niu J Banbury MK Zhou L Taylor DO Starling RC Influence of different implantation techniques on long-term survival after orthotopic heart transplantation: an echocardiographic study. *J Heart Lung Transplant.* (2007) 26:1243–8. 10.1016/j.healun.2007.09.016 18096474

[19] Lower RR Shumway NE. Studies on orthotopic homotransplantation of the canine heart. *Surg Forum.* (1960) 11:18–9. 13763847

[20] Crumbley AJ III Van Bakel AB. Tricuspid valve repair for biopsy-induced regurgitation after cardiac transplantation. *Ann Thorac Surg.* (1994) 58:1156–60. 10.1016/0003-4975(94)90478-2 7944770

[21] Filsoufi F Salzberg SP Anderson CA Couper GS Cohn LH Adams DH. Optimal surgical management of severe tricuspid regurgitation in cardiac transplant patients. *J Heart Lung Transplant.* (2006) 25:289–93. 10.1016/j.healun.2005.09.013 16507421

[22] Wong RC Abrahams Z Hanna M Pangrace J Gonzalez-Stawinski G Starling R Tricuspid regurgitation after cardiac transplantation: an old problem revisited. *J Heart Lung Transplant.* (2008) 27:247–52. 10.1016/j.healun.2007.12.011 18342744

[23] Berger Y Har Zahav Y Kassif Y Kogan A Kuperstein R Freimark D Tricuspid valve regurgitation after orthotopic heart transplantation: prevalence and etiology. *J Transplant.* (2012) 2012:120702. 10.1155/2012/120702 23097690 PMC3477771

[24] Kwon MH Shemin RJ. Tricuspid valve regurgitation after heart transplantation. *Ann Cardiothorac Surg.* (2017) 6:270–4. 10.21037/acs.2017.04.02 28706871 PMC5494414

[25] Sherman-Weber S Axelrod P Suh B Rubin S Beltramo D Manacchio J Infective endocarditis following orthotopic heart transplantation: 10 cases and a review of the literature. *Transpl Infect Dis.* (2004) 6:165–70. 10.1111/j.1399-3062.2004.00074.x 15762934

[26] Misumida N Steidley DE Eleid MF. Edge-to-edge tricuspid valve repair for severe tricuspid regurgitation 20 years after cardiac transplantation. *ESC Heart Fail.* (2020) 7:4320–5. 10.1002/ehf2.12992 32945151 PMC7754756

[27] Cavero MA Pulpon LA Rubio JA Burgos R Lozano I Moreu J Mitral valve replacement in a heart transplant recipient with iatrogenic mitral regurgitation. *Ann Thorac Surg.* (1996) 61:1530–2. 10.1016/0003-4975(95)01178-1 8633978

[28] Wijburg ER Balk AH van Herwerden LA. Double valve repair in a transplanted heart. *J Thorac Cardiovasc Surg.* (1998) 115:250–1. 10.1016/s0022-5223(98)70469-6 9451075

[29] Wigfield CH Lewis A Parry G Dark JH. Mitral valve dysfunction and repair following orthotopic heart transplantation: a case report. *Transplant Proc.* (2008) 40:1796–7. 10.1016/j.transproceed.2007.10.010 18589200

[30] Depace NL Rohrer AH Kotler MN Brezin JH Parry WR. Rapidly progressing, massive mitral annular calcification. Occurrence in a patient with chronic renal failure. *Arch Intern Med.* (1981) 141:1663–5. 7305574

[31] Joyce DL Russell SD Conte JV Cattaneo SM. Aortic valve replacement in a diseased bicuspid valve eleven years after transplantation. *Interact Cardiovasc Thorac Surg.* (2009) 8:594–5. 10.1510/icvts.2008.194050 19223305

[32] Goenen MJ Jacquet L De Kock M Van Dyck M Schoevardts JC Chalant CH. Aortic valve replacement thirty-one months after orthotopic heart transplantation. *J Heart Lung Transplant.* (1991) 10:604–7. 1911806

[33] Fiane AE Svennevig JL Froysaker T. Aortic valve replacement four years after cardiac transplantation. *Eur Heart J.* (1993) 14:1140–2. 10.1093/eurheartj/14.8.1140 8404946

[34] Unic D Starcevic B Sicaja M Baric D Rudez I Biocic S Aortic valve endocarditis in a transplanted heart after urethral instrumentation. *Ann Thorac Surg.* (2013) 96:e61–2. 10.1016/j.athoracsur.2013.03.113 23992731

[35] Pozzi M Hanna S Sebbag L Obadia JF. Donor aortic dissection in a heart transplantation recipient. *Interact Cardiovasc Thorac Surg.* (2018) 27:790–1. 10.1093/icvts/ivy153 29718266

[36] Vigano M Rinaldi M D’Armini AM Pederzolli C Minzioni G Grande AM. The spectrum of aortic complications after heart transplantation. *Ann Thorac Surg.* (1999) 68:105–11. 10.1016/s0003-4975(99)00471-3 10421124

[37] Gao SZ Alderman EL Schroeder JS Silverman JF Hunt SA. Accelerated coronary vascular disease in the heart transplant patient: coronary arteriographic findings. *J Am Coll Cardiol.* (1988) 12:334–40. 10.1016/0735-1097(88)90402-0 3292629

[38] Musci M Pasic M Meyer R Loebe M Wellnhofer E Weng Y Coronary artery bypass grafting after orthotopic heart transplantation. *Eur J Cardiothorac Surg.* (1999) 16:163–8. 10.1016/s1010-7940(99)00207-9 10485415

[39] Bhama JK Nguyen DQ Scolieri S Teuteberg JJ Toyoda Y Kormos RL Surgical revascularization for cardiac allograft vasculopathy: is it still an option? *J Thorac Cardiovasc Surg.* (2009) 137:1488–92. 10.1016/j.jtcvs.2009.02.026 19464469

[40] Prada-Delgado O Estevez-Loureiro R Lopez-Sainz A Gargallo-Fernandez P Paniagua-Martin MJ Marzoa-Rivas R Percutaneous coronary interventions and bypass surgery in patients with cardiac allograft vasculopathy: a single-center experience. *Transplant Proc.* (2012) 44:2657–9. 10.1016/j.transproceed.2012.09.043 23146485

[41] Lopes RD Mehta RH Hafley GE Williams JB Mack MJ Peterson ED Project of Ex Vivo vein graft engineering via transfection IVI. Relationship between vein graft failure and subsequent clinical outcomes after coronary artery bypass surgery. *Circulation.* (2012) 125:749–56. 10.1161/CIRCULATIONAHA.111.040311 22238227 PMC3699199

[42] Tchana-Sato V Ancion A Ansart F Defraigne JO. Constrictive pericarditis after heart transplantation: a case report. *Eur Heart J Case Rep.* (2020) 4:1–6. 10.1093/ehjcr/ytaa240 32974473 PMC7501930

[43] Umer A Khalid N Chhabra L Memon S Spodick DH. Constrictive pericarditis complicating cardiac transplantation. *J Cardiothorac Surg.* (2015) 10:109. 10.1186/s13019-015-0314-x 26302865 PMC4549104

[44] Kumar R Entrikin DW Ntim WO Carr JJ Kincaid EH Hines MH Constrictive pericarditis after cardiac transplantation: a case report and literature review. *J Heart Lung Transplant.* (2008) 27:1158–61. 10.1016/j.healun.2008.07.010 18926409

