# Supplementary material for: Indications, Complications, and Outcomes of Cardiac Surgery After Heart Transplantation: Results From the Cash Study
Source: Front Cardiovasc Med. 2022 Jun 9;10:879612. doi: 10.3389/fcvm.2022.879612 (PMC9218180; doi:10.3389/fcvm.2022.879612)
Supplement: Supplementary file 1 [file Table_1.docx]

**Indications, Complications, and Outcomes of Cardiac Surgery after Heart Transplantation: Results from the CASH Study**

**Supplemental Material**
